# The Clinical Application of Double Taylor Spatial Frame in Segmental Tibial Fracture

**DOI:** 10.1111/os.14045

**Published:** 2024-04-25

**Authors:** Qi‐Jun Zhao, Zhao Liu, Xun Sun, Ning‐Ning Zhang, Wei‐Guo Xu, Tao Zhang

**Affiliations:** ^1^ Clinical College of Orthopedics Tianjin Medical University Tianjin China; ^2^ Department of Orthopedics Sinopharm North Hospital Baotou China; ^3^ Department of Orthopaedics Ward One Tianjin Hospital Tianjin China

**Keywords:** External fixation, Hip–knee–ankle angle, Johner–Wruhs tibial fracture outcome criteria, Segmental tibial fracture, Taylor Spatial Frame

## Abstract

**Objectives:**

Multi‐planar external fixation has been used for the management of segmental tibial fractures with severe soft tissue injuries. However, fewer specialized studies have been reported. The primary aim of this study was to describe our experience of treating fractures of this type using the Taylor Spatial Frame and Ilizarov external fixation methods.

**Methods:**

We retrospectively analyzed 33 patients with segmental tibial fracture treated at our institution between January 2016 and December 2020. The patients were divided into double Taylor Spatial Frame (D‐TSF) and Ilizarov groups based on the external fixation structure. Baseline demographic data included sex, age, injury side and cause, open or closed fracture, time from injury to surgery, complications, and external frame removal and fracture healing time. The hip–knee–ankle angle (HKA) was measured from preoperative, immediate postoperative, and final follow‐up full‐length X‐rays of bilateral lower limbs. We determined the degree of deviation in the HKA by calculating the difference between the measured angle and the ideal value of 180°; the absolute value was used to assess recovery of the lower limb force line. At the final follow‐up, Johner–Wruhs tibial fracture outcome criteria (J‐W TFOC) were used to classify the postoperative function of the affected limb as excellent, good, moderate, or poor. Count data were analyzed with the chi‐square test or Fisher's exact test; the Mann–Whitney *U* test was used for rank data.

**Results:**

No statistically significant differences were observed between the two groups in terms of sex, age, side of injury, cause of injury, closed or open fracture, or time between injury and surgery, which indicates that the groups were comparable (*p* > 0.05). A statistically significant difference was observed in external frame removal and fracture healing time between the D‐TSF and Ilizarov groups (36.24 ± 8.34 *vs* 45.42 ± 10.21 weeks, *p* = 0.009; 33.33 ± 8.21 *vs* 42.00 ± 9.78 weeks, *p* = 0.011). The Johner–Wruhs criteria were used to assess the function of the affected limb, the D‐TSF group performed better in correcting the lower limb force line than the Ilizarov group. A statistically significant difference in terms of excellent ratings was observed between the two groups (18/2/1/0 *vs* 5/5/1/1, *p* = 0.010). Postoperative follow‐up X‐rays demonstrated a significant improvement in the HKA in both groups immediately after surgery and at the final follow‐up compared to the angle before surgery. At the final follow‐up, a statistically significant difference was observed in the degree of deviation in the HKA between the two groups (1.58° ± 0.84° *vs* 2.37° ± 1.00°, *p* = 0.023).

**Conclusion:**

The D‐TSF treatment is associated with minimal secondary damage to soft tissue, a straightforward and minimally invasive procedure, multiplanar stable fracture fixation, and optimization of fracture alignment and lower limb force lines, therefore, it is highly effective therapeutic option for segmental tibial fracture.

## Graphical abstract

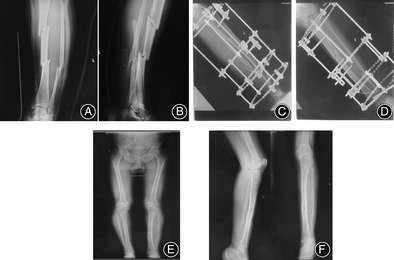

## Introduction

Segmental fractures of the tibia, which are characterized by the presence of ≥ 2 fracture lines in the tibial shaft with a free middle segment, account for 3%–12% of total body fractures.^1^, ^2^ These fractures are caused by high‐energy trauma and are classified as AO stage 42‐C2. The anteromedial aspect of the tibia lacks muscle tissue, which renders fractures in this area highly vulnerable to soft tissue injury. Hence, open fractures of the tibia often occur in combination with significant soft tissue decortication or skin defects, accounting for 24% of tibial fractures.^3^, ^4^

Segmental tibial fractures are difficult to treat usually because of the associated soft tissue injuries and poor outcomes with conservative management. Therefore, surgery is the preferred management option—it emphasizes early reduction and fixation of the fracture while preserving the surrounding soft tissue to minimize secondary surgical injury.^5^, ^6^ Giannoudis *et al*. studied 27 segmental tibial fracture fixations and revealed that high‐energy segmental fractures can disrupt blood supply of the free fracture segment, resulting in non‐union and delayed healing.^7^ These complications highlight the importance of early stabilization and fixation to prevent further damage and promote successful healing. Giotakis *et al*. demonstrated that segmental tibial fractures present a significant challenge to achieving multiplanar stable fixation of the fixation device.^8^ The treatment of fixation method should be based on high‐energy damage inflicted on the soft tissue and the relatively increased risk for infection in open fractures. Effective management strategies, such as timely administration of antibiotics, thorough wound debridement, and early soft tissue coverage, are essential in reducing the risk for complications.^9^, ^10^

Optimal fixation for segmental tibial fracture, such as plating, intramedullary nailing, and external fixation, remain controversial. Therefore, the choice of surgery should be made after careful consideration of the state of the soft tissue in the affected limb.^6^ Traditional internal fixation methods depend on the local soft tissue conditions and may result in various postoperative complications. By contrast, external fixation is a minimally invasive, safe, and effective alternative that avoids secondary damage to the soft tissue, preserves blood flow to the fracture site, and is preferred by many orthopedic surgeons.

The Taylor Spatial Frame (TSF) is external fixator evolved from the Ilizarov circular frame, comprising two fixation rings (full or partial) connected by six length‐adjusted universal support struts, and is a unique six‐axis synergistic linkage system that allows for a high degree of adjustability and simultaneous correction of multiplanar deformities while maintaining the minimal invasiveness and ease of operation of the original external fixation frame. The TSF features specialized software that analyzes postoperative measurements of fracture deformity and external fixation parameters. So, this allows for personalized electronic prescriptions possible, which can accurately align the fracture end and adjust the lower limb force line.^11^, ^12^

Although the TSF is commonly used for tibial fracture fixation, its use in segmental tibial fracture is less. This study collected and analyzed the clinical data for patients undergoing D‐TSF and Ilizarov surgical procedures for segmental tibial fracture treatments. We aimed at: (i) elucidating on the surgical procedures and operation skills of D‐TSF for segmental tibial fracture treatments; and (ii) analyzing and comparing surgical procedures and clinical effects of D‐TSF and Ilizarov in segmental tibial fracture treatment.

## Methods

### Patients

Patients were included in the study if they met the following criteria: (i) fracture classified as AO stage 42‐C2; (ii) age 18–65 years; and (iii) treatment with TSF or Ilizarov external fixation frame. The exclusion criteria were: (i) patients with proximal and distal tibial articular surface and Gustilo type IIIC fractures, pathological fractures, or psychiatric disorders; (ii) patients who demonstrated poor compliance; and (iii) patients were unable to wear external fixation frame were excluded. Patients underwent TSF or Ilizarov external fixation (Tianjin Xinzhong Medical Equipment, Tianjin, China). The Medical Ethics Committee of Tianjin Hospital approved the study protocol (2023 Medical Ethics Review 107), and all patients signed written informed consent prior to their inclusion in this study.

We retrospectively analyzed 33 patients with segmental tibial fracture treated at our institution between January 2016 and December 2020. The study population included 23 males and 10 females ages 23–65 (mean age: 45.2 ± 11.0) years. Of these patients, 21 had left‐sided fractures and 12 had right‐sided fractures. Factors that contributed to the fractures included traffic accidents (72.73%), injuries from bruises (18.18%), and falls (9.09%). The patients were divided into D‐TSF and Ilizarov groups based on the external fixation structure, with 21 patients in the D‐TSF group and 12 in the Ilizarov group.

In addition, 17 patients had closed fractures and 16 had open fractures. Soft tissue injuries were classified according to criteria of Gustilo *et al*.^13^ In general, Type III fractures had open wounds larger than 10 cm or fracture patterns with segmental comminution. Within this category, Type IIIA fractures were those that could be closed, either by delayed primary closure or split thickness skin graft, implying adequate soft tissue coverage of bone. Type IIIB fractures were those identified after initial debridement and stabilization which required rotational flap for soft tissue coverage. Tables 1 and 2 provide the clinical details of the soft tissue injury of the Ilizarov group and the D‐TSF group, respectively.

**TABLE 1:** Clinical details of soft tissue injury of the Ilizarov group

Case number	Open/closed	Cause of injury	Associated injury	Coverage procedures
1	Open, IIIA	Bruise	SSTSWB, skin bruise	Primary closure
2	Closed	Traffic accident	SSTSWB, skin bruise	‐
3	Closed	Traffic accident	SSTSWB, skin bruise	‐
4	Open, IIIA	Fall	SSTSWB	Primary closure
5	Closed	Traffic accident	SSTSWB, skin bruise	‐
6	Closed	Bruise	SSTSWB	‐
7	Open, IIIA	Traffic accident	SSTSWB, skin bruise	Primary closure
8	Closed	Fall	SSTSWB, skin bruise	‐
9	Open, IIIA	Traffic accident	SSTSWB, skin bruise	Primary closure
10	Open, IIIA	Traffic accident	SSTSWB, small skin defect	Primary closure + VSD
11	Open, IIIA	Bruise	SSTSWB, small skin defect	Primary closure + VSD
12	Closed	Traffic accident	SSTSWB	‐

Abbreviations: SSTSWB, Severe soft tissue swelling with blister; VSD, Vacuum sealing drainage.

**TABLE 2:** Clinical details of soft tissue injury of double Taylor Spatial Frame group

Case number	Open/Closed	Cause of injury	Associated injury	Coverage procedures
1	Closed	Traffic accident	SSTSWB, skin bruise	‐
2	Open, IIIA	Traffic accident	SSTSWB, small skin defect	Primary closure + VSD
3	Open, IIIA	Traffic accident	SSTSWB, small skin defect	Primary closure + VSD
4	Closed	Fall	SSTSWB, skin bruise	‐
5	Open, IIIA	Bruise	SSTSWB	Primary closure
6	Closed	Traffic accident	SSTSWB, skin bruise	‐
7	Closed	Traffic accident	SSTSWB, skin bruise	‐
8	Open, IIIA	Fall	SSTSWB	Primary closure
9	Closed	Traffic accident	SSTSWB, skin bruise	‐
10	Closed	Bruise	SSTSWB, skin bruise	‐
11	Open, IIIA	Traffic accident	SSTSWB	Primary closure
12	Closed	Fall	SSTSWB	‐
13	Open, IIIA	Traffic accident	SSTSWB	Primary closure
14	Open, IIIA	Traffic accident	SSTSWB, small skin defect	Primary closure + VSD
15	Open, IIIA	Bruise	SSTSWB, small skin defect	Primary closure + VSD
16	Closed	Traffic accident	SSTSWB, skin bruise	‐
17	Open, IIIA	Traffic accident	SSTSWB, small skin defect	Primary closure + VSD
18	Closed	Bruise	SSTSWB, skin bruise	‐
19	Closed	Traffic accident	SSTSWB, skin bruise	‐
20	Open, IIIA	Bruise	SSTSWB, small skin defect	Primary closure + VSD
21	Closed	Traffic accident	SSTSWB, skin bruise	‐

Abbreviation: SSTSWB, severe soft tissue swelling with blister; VSD, vacuum sealing drainage.

### Preoperative Preparation

Upon admission, the patients with open fractures underwent debridement and suturing of the affected limb whilst in heel traction in the emergency operating room. Vacuum sealing drainage (VSD) was used to cover wounds caused by open fractures that could not be closed with sutures. Next, the patient was admitted to our department, where routine preoperative investigations, including laboratory tests and imaging of the affected limb, were conducted to rule out contraindications for surgery. Preoperative anticoagulants administered to prevent lower limb deep vein thrombosis were discontinued 24 h before surgery.

### Surgical Method

All surgeries were performed under epidural anesthesia by the same surgical team specializing in tibial surgery via external fixation.

In the D‐TSF group, the TSF was preassembled using a 2/3 ring for the proximal bone segment and full rings for the middle and distal bone segments. The middle ring was positioned based on the middle bone segment location, whereas the proximal and distal rings were placed parallel to the knee and ankle joint surfaces, respectively; the three rings were connected with six rapid support struts. To ensure that the half‐pins (5 mm diameter) were perpendicular to the fracture segment, the proximal and distal rings were each secured with three half‐pins and the middle ring was secured with two half‐pins. After each fracture segment was fixed, it was closed repositioned individually by adjusting the length of the support struts based on the fracture type; simple fractures were addressed first, followed by complex ones. Any residual deformities were addressed postoperatively.

In the Ilizarov group, the Ilizarov external fixation frame was preassembled with full rings connected by threaded struts. After confirming the position of the middle segment fracture, the middle ring was positioned by adjusting the length of the four threaded struts at the distal and proximal ends. Next, the tibial fracture end was repositioned and secured with manual or point repositioning forceps and kerf pins. The proximal, intermediate, and distal rings were fitted vertically to the bone segment. Olive pins and half‐pins were threaded separately through each fracture segment; every two full rings were fixed with three or four threaded struts of different lengths connected to one another. Intraoperative fluoroscopy confirmed the alignment of the fracture ends; the olive pins were tensioned, and then, the three or four full rings were fixed before the threaded struts were locked.

### Postoperative Management

**FIGURE 1:**
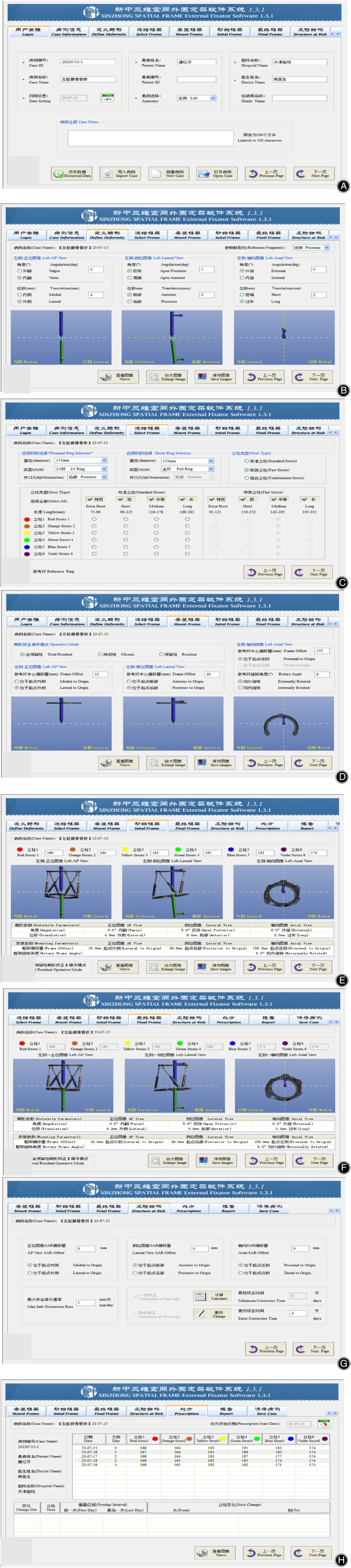
Specific steps for generating electronic prescriptions with supporting software (A) After successful login, enter the patients name, injured limb (left or right), consultation hospital, attending physician and other basic information. (B) Select the reference bone segment (proximal or distal) and enter the six deformity parameters obtained from the postoperative lower limb full‐length X‐Ray measurements, the program software will automatically provide corresponding simulated image, showing the fracture deformity in orthotropic, lateral, and axial positions. (C) Enter three frame parameters such as the ring type and diameter of the proximal and distal rings and the type of support struts. (D) Select the total residual deformity operative mode, enter four mounting parameters corresponding to the position of the reference ring at the point of origin and confirm the relationship between the reference ring and the reference bone segment through the generated simulated images. (E) Enter the length of each support strut, the program software will automatically display the initial TSF simulated images installed on the injured limb. (F) The program software gives the final length of each support strut required to correct the deformity and display the final TSF simulated images. (G) Enter the coordinates of the structure at risk when the deformity is corrected and the max safe distraction rate. (H) The program software gives the number of days required to correct the deformity and the value of the correction per day for each support struts.

In both groups, needle tracts were routinely dressed and sterilized postoperatively to prevent infection. On postoperative day 1, full‐length X‐rays of bilateral lower limbs were obtained to evaluate significant angulation, displacement, and rotational deformity. In the D‐TSF group, three groups of parameters (i.e., deformity, mounting and frame parameters) were measured and an electronic prescription was generated using the application software (version 1.3.1; Xinzhong Medical Devices Co., Ltd., Tianjin, China), which is used by doctors to correct the residual deformities by adjusting the length of struts daily according to the patient's actual condition. (The specific steps for generating electronic prescriptions with supporting software were shown in Figure 1). In the Ilizarov group, a hinge or traverse device was used to correct the residual deformity. The length of the threaded strut was adjusted to apply pressure to the fracture ends to promote healing. On postoperative day 2, patients were instructed to begin functional rehabilitation training of the knee and ankle joints to prevent joint stiffness and deep vein thrombosis. Antibiotics were administered for 3 days postoperatively. Patients in the D‐TSF and Ilizarov groups were instructed to use double crutches and perform weightless exercises for functional rehabilitation of the affected limb for 2 weeks postoperatively. At approximately 12 weeks after surgery, weight‐bearing status during walking was assessed based on the bone healing status.

### Follow‐up and Outcome Assessment

After discharge, the patients were reexamined at an interval of 4 weeks until the fracture healed and the external fixator was removed. During regular follow‐ups, X‐rays were taken to monitor the fracture alignment and bone healing; the external fixation frame was adjusted if necessary. In the D‐TSF group, the axial load‐sharing ratio was evaluated in the third month after surgery. When the load‐sharing ratio was < 10%, a comprehensive evaluation of fracture healing was performed based on clinical and imaging findings for the affected limb, followed by a mock removal of the frame. The external frame was removed after 2 weeks if the patients did not experience any discomfort.^14^ Although patients in the Ilizarov group received the same treatment protocol as those in the D‐TSF group, they were not subjected to this specific test.

Baseline demographic data included sex, age, injury side and cause, open or closed fracture, time from injury to surgery, complications, and external frame removal and fracture healing time. The hip–knee–ankle angle (HKA) was measured from preoperative, immediate postoperative, and final follow‐up full‐length X‐rays of bilateral lower limbs. We determined the degree of deviation in the HKA by calculating the difference between the measured angle and the optimal value of 180°; the absolute value was used to assess recovery of the lower limb force line.^15^ At the final follow‐up, the Johner–Wruhs tibial fracture outcome criteria were used to classify the postoperative function of the affected limb as excellent, good, moderate, or poor. We calculated an excellent rating using the following formula: (excellent + good)/total number of cases × 100%.

### Statistical Analyses

Statistical analyses were performed in SPSS (version 23.0; IBM, Armonk, NY, USA). Quantitative data are presented as means ± standard deviations. For normally distributed data, the independent‐samples *t*‐test was used for comparisons of the two groups, whereas the paired *t*‐test was used for comparisons within groups at different time points. The rank sum test was used for non‐normally distributed data. Count data were analyzed with the chi‐square test or Fisher's exact test; the Mann–Whitney *U* test was used for rank data. *p* < 0.05 was considered statistically significant.

## Results

### Demographic Data

Patients in the D‐TSF and Ilizarov groups were followed for a median of 14 (6–22) months. No statistically significant differences were observed between the two groups in terms of sex, age, side of injury, cause of injury, closed or open fracture, or time between injury and surgery, which indicates that the groups were comparable (*p* > 0.05; Table 3).

**TABLE 3:** Comparison of preoperative information between the D‐TSF and Ilizarov groups

Variables	Ilizarov (*n* = 12)	D‐TSF (*n* = 21)	*p*
Age (years, mean ± SD)	49.75 ± 8.56	42.52 ± 11.56	>0.05
Sex (*n*, male/female)	8/4	15/6	>0.05
Time from injury to surgery (days, mean ± SD)	9.42 ± 3.50	7.52 ± 3.17	>0.05
Injury side (*n*, left/right)	7/5	14/7	>0.05
Cause of injury (*n*, traffic accidents, bruises, or falls)	7/3/2	13/5/3	>0.05
Closed or open	6/6	11/10	>0.05

Abbreviation: D‐TSF, double Taylor Spatial Frame; Ilizarov, Ilizarov frame; SD, standard deviation.

### Surgical Results

All fractures in both groups eventually healed. The external frame removal and fracture healing time differed significantly between the D‐TSF and Ilizarov groups (*p* < 0.05; Table 4). At the final follow‐up, all patients were evaluated against the Johner–Wruhs criteria to assess the function of the affected limb. The results showed an overall excellent rating of 90.91%, with 23 cases rated as excellent, seven as good, two as moderate, and one as poor. The excellent rating was 95.24% in D‐TSF group, with 18 rated as excellent, two as good, and one as moderate. By contrast, the excellent rating was 83.33% in the Ilizarov group, with five cases rated as excellent, five as good, one as moderate, and one as poor. A statistically significant difference in terms of excellent ratings was observed between the two groups (*p* < 0.05). Postoperative follow‐up X‐rays demonstrated a significant improvement in the HKA in both groups immediately after surgery and at the final follow‐up compared to the angle before surgery. Furthermore, no statistically significant differences were observed between the two postoperative time points (*p* > 0.05). At the final follow‐up, a statistically significant difference was observed in the degree of deviation in the HKA between the two groups (*p* < 0.05; Table 5).

**TABLE 4:** Comparison of FH and EFR time between the D‐TSF and Ilizarov groups (means ± SD)

Groups	N	FH (weeks)	EFR (weeks)
D‐TSF	21	33.33 ± 8.21	36.24 ± 8.34
Ilizarov	12	42.00 ± 9.78	45.42 ± 10.21
Statistical value		*t* = 2.723 *p* = 0.011	*t* = 2.803 *p* = 0.009

Abbreviations: D‐TSF, double Taylor Spatial Frame; EFR, external frame removal; FH, fracture healing; Ilizarov, Ilizarov frame; SD, standard deviation.

**TABLE 5:** Comparison of J‐W TFOC of the affected limb and deviation of HKA angle at preoperative, IP, and FFU in the D‐TSF and Ilizarov groups

Groups	N	J‐W criteria	Deviation of HKA (°)		
			Preoperative	IP	FFU
D‐TSF	21	18/2/1/0	9.15 ± 2.39	3.00 ± 1.94	1.58 ± 0.84
Ilizarov	12	5/5/1/1	11.03 ± 3.71	3.81 ± 2.53	2.37 ± 1.00
Statistical value		*p =* 0.010	*t* = 1.772 *p*>0.05	*t* = 1.032 *p*>0.05	*t* = 2.398 *p* = 0.023

Abbreviations: D‐TSF, double Taylor Spatial Frame; FFU, Final follow‐up; HKA, hip–knee–ankle angle; IP, immediate postoperative; Ilizarov, Ilizarov frame; J‐W TFOC, Johner‐Wruhs tibial fracture outcome criteria.

### Complications

In our study, early needle tract infection occurred in only 12 patients, usually at 2 weeks postoperatively, with a total infection rate of 12/33 = 36.36%, including seven cases in the D‐TSF group (infection rate 7/21 = 33.33%) and five cases in the Ilizarov group (infection rate 5/12 = 41.67%). There were no cases of deep infections, and all cases of needle tract site infections resolved successfully after aseptic dressing change and short‐term oral antibiotics, with no statistically significant difference (*χ* ^2^ = 0.23, *p* > 0.05). No serious complications such as osteomyelitis, delayed healing and non‐healing occurred.

**FIGURE 2:**
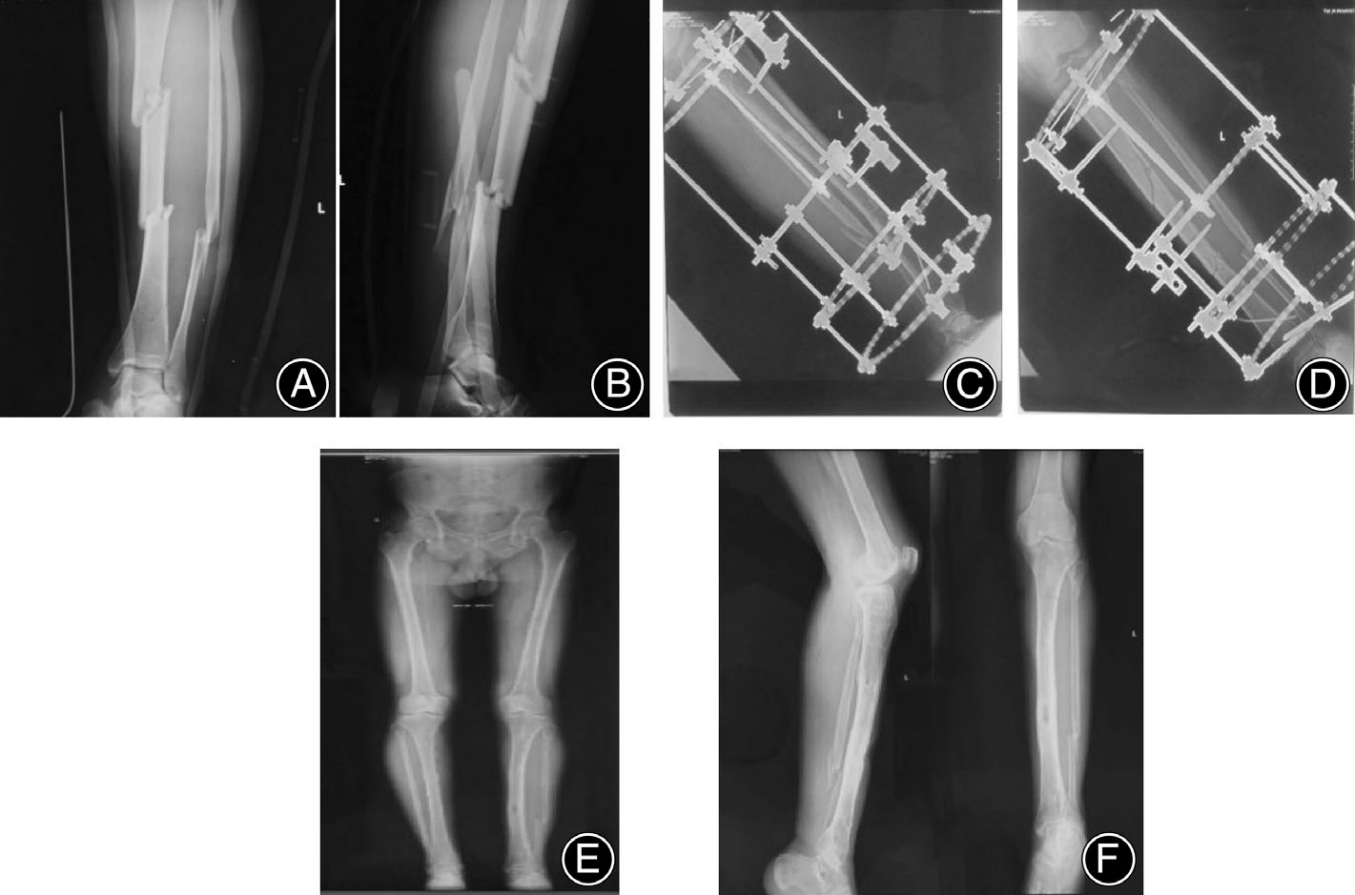
A 47‐year‐old male patient with left segmental tibial fracture was treated with Ilizarov frame. (A and B) Preoperative tibiofibular X‐ray imaging showed left segmental tibial fracture. (C and D) Immediate postoperative full‐length tibiofibular X‐ray imaging showed that Ilizarov frame fixation position of the fracture was good. (E) After Ilizarov frame removal, full‐length X‐rays of bilateral lower limbs showed good force lines. (F) After Ilizarov frame removal, full‐length tibiofibular X ray showed fracture healing with micro‐deformity.

**FIGURE 3:**
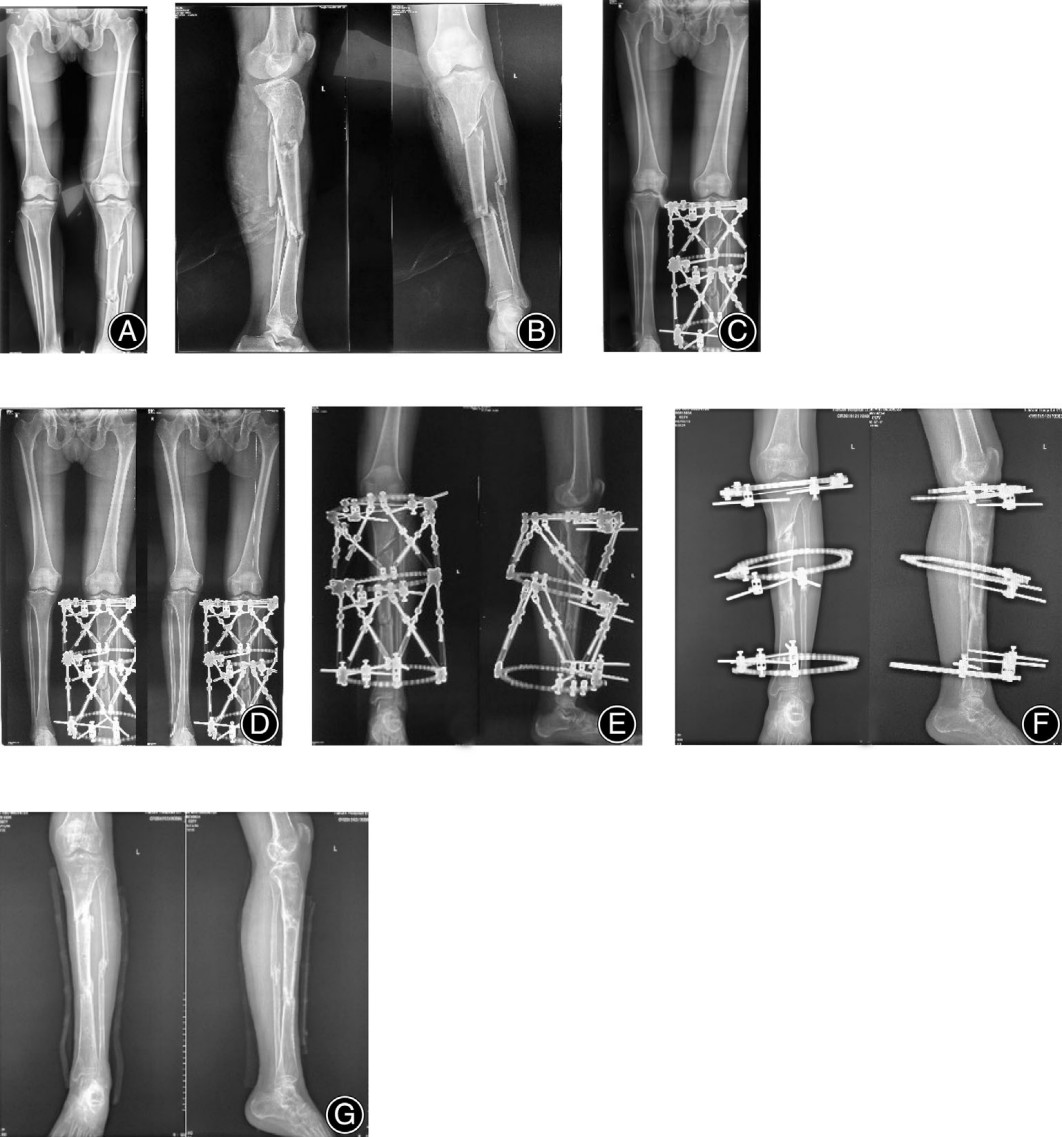
A 36‐year‐old male patient with left segmental tibial fracture was treated with double Taylor Spatial Frame (DTSF). (A) Preoperative full‐length X‐rays of bilateral lower limbs showed poor force lines. (B) Preoperative full‐length tibiofibular X‐ray imaging showed left segmental tibial fracture. (C) Immediate postoperative full‐length X‐rays of bilateral lower limbs showed better force lines and that D‐TSF frame fixation position of the fracture had residual deformities. (D and E) After software corrected, full‐length X‐rays of bilateral lower limbs showed even better force lines and that D‐TSF frame fixation position of the fracture residual deformities were corrected. (F) Just before D‐TSF frame removal, full‐length tibiofibular X‐Ray showed fracture healing with less micro‐deformity. (G) After D‐TSF removal, full‐length tibiofibular X‐Ray showed fracture healing with less microdeformity.

Typical cases were shown in Figures 2 and 3.

## Discussion

Our research demonstrates that TSF was different in that it had good mechanical and biological properties, more scientific frame structure design, convenient use during surgery, and precise reduction of fractures and severe deformities with the help of supporting application software after surgery, which could adjust the overall force line of the affected limb. Moreover, patients could perform functional rehabilitation training with early weight‐bearing, thus accelerating fracture healing and functional recovery of the affected limb, and ultimately shortening the time required for fracture healing and frame‐wearing. We admit that TSF is more complex and expensive than Ilizarov, but this is only temporary. With the development of orthopedic deformity correction techniques and technology, the cost of computer software‐assisted TSF will definitely decrease. And, most importantly, the final clinical results obtained from the treatment of segmental tibial fractures with Ilizarov are not satisfactory. Considering the ultimate benefit of the patient, it is a better choice to preferentially use TSF to treat segmental tibial fractures when their economic situation can bear it.^16^

### Fixation Principles and Surgical Procedures of D‐TSF

In our current study, we designed and used D‐TSF to treat tibial segmental fractures. First, during the surgical procedure, after the D‐TSF was installed and fixed, the operator only needed to adjust the proximal and distal fracture segments with the help of an assistant by adopting closed reduction method, that is, it was not necessary to use open reduction to restore the fracture end to the right position and alignment, which avoided secondary surgical damage caused by the open reduction to the skin and soft tissues of affected limb that were swollen and the blood supply of the fracture end. Second, the diameter of the half pin used in D‐TSF fracture fixation was only 5 mm, which caused very little damage to the skin and soft tissue. Furthermore, D‐TSF could correct the deformities of fracture end after surgery without changing the external frame structure again, which reduced the number of surgeries and the chance of secondary surgical damage to the skin and soft tissues accordingly. In conclusion, D‐TSF treatment of segmental tibial fracture is less traumatic and has less secondary surgical damage to the affected limb. Liu *et al*. used TSF to treat high‐energy tibial fractures and demonstrated its potential to facilitate precise three‐dimensional correction in complex and severe deformities, in particular those with extensive soft tissue damage where internal fixation is not feasible.^17^ The TSF, a computer‐assisted multiplanar simultaneous orthopedic system, which is essentially a parallel robot, has significantly improved imaging and clinical outcomes in severe tibiofibular fracture fixation. In the terminology of its application, the bone segment that remains fixed and motionless as a reference is called reference bone segment, while the other bone segment that is considered to produce angular, translational, and rotational changes relative to the reference bone segment is called mobile bone segment. During the TSF deformity correction process, the mobile bone segment produces passive activity relative to the reference bone segment to obtain deformity correction. During its use, the TSF computer‐assisted software program requires input of 13 parameters, 10 of which (i.e., six deformity parameters and four mounting parameters) are related to the specific location of the deformity, thus commanding one bone segment to produce displacement and angulation relative to the other, and ultimately the two segments achieve satisfactory alignment, registration and length restoration. We optimized the TSF based on individual fracture characteristics to provide intraoperative multiplanar stabilization of different fracture segments, allowing for segmental fracture repositioning and lower limb force line adjustment. Furthermore, the mechanical load test of the external frame guides patients' rehabilitation exercises and subsequent frame removal, leading to precise, comprehensive, and intelligent fracture treatment.^14^, ^15^, ^18^, ^19^, ^20^, ^21^

### Effectiveness of D‐TSF for Segmental Tibial Fracture

In our retrospective study, we evaluated the effectiveness of D‐TSF for segmental tibial fracture fixation. Our findings revealed a mean fracture healing time of (33.33 ± 8.21) weeks and a mean external frame removal time of (36.24 ± 8.34) weeks, consistent with previous reports. In addition, Teraa *et al*. revealed an average healing time of 34 weeks for segmental tibial fractures; these fractures heal slowly and have more complications than nonsegmental tibial fractures.^1^ Therefore, such fractures require specialized management in a trauma center. Similarly, Tucker *et al*. used TSF to fix high‐energy open tibial fractures and revealed a mean fracture healing time of (235 ± 183) days (about 34 weeks) and a mean frame removal time of (206.7 ± 149.4) days (about 29 weeks).^19^ O'Neill *et al*. conducted a study in Dublin using an external ring fixator for lower limb fracture and revealed a healing time of 230 days (about 33 weeks) for tibial fractures.^22^ However, they did not differentiate healing times for segmental tibial fractures. We found that patients in the D‐TSF group demonstrated significantly shorter fracture healing and external frame removal times compared to the other patients (36.48 ± 9.64 *vs* 39.58 ± 9.97 weeks); this improvement can be attributed to the TSF axial load‐sharing ratio test conducted in the third month after surgery. This test evaluates the stiffness of the bone calculus quantitatively and releases the stress on the fracture break end by loosening the six support struts of the TSF, as a result, local stress shielding is reduced, which promotes fracture healing and significantly shortens the frame removal time.^14^

In addition, the 16 open fractures had fracture healing times of (38.19 ± 11.26) and external frame removal times of (41.19 ± 11.56) weeks; the 17 closed fractures had fracture healing times of (34.88 ± 7.83) and external frame removal times of (38.06 ± 8.28) weeks. No statistically significant differences were observed between the two groups, which indicates that segmental tibial fractures have similar healing times regardless of whether they are open or closed. These findings are consistent with a study conducted by Menakaya *et al*.^23^

As for recovery of the lower limb force line, we had found in clinical practice that, compared with Ilizarov, although both of them could stabilize and fix segmental tibial fracture in multiple planes, D‐TSF could reposition the fracture ends and adjust the lower limb force line during and after surgery, and its unique six‐axis synergistic linkage system not only could correct fracture deformity by segments and steps, but also could correct various multiangle and multiplane deformities at the same time, and the residual deformity of the fracture could still be further corrected with assistance of the computer program software after surgery. More importantly, as mentioned previously, D‐TSF could correct deformities without changing frame structure one more time. In contrast, the ability of Ilizarov for segmental tibial fracture deformity to be adjusted simultaneously in multiangle and multiplane was poor, and sometimes a second surgery was needed to add a hinge or a traverse device to correct the residual deformity. The HKA improved both significantly in the D‐TSF and Ilizarov groups immediately after surgery and at the final follow‐up, which was more obvious in the D‐TSF group. However, a statistically significant difference was observed between the two groups in terms of the degree of deviation in the HKA at the final follow‐up, which indicates that D‐TSF group performed better in correcting the lower limb force line. Furthermore, patients were evaluated at the final follow‐up using the Johner–Wruhs tibial fracture outcome criteria to assess the function of the affected limb. The results showed an overall excellent rating of 90.91*%*, with 23 cases rated as excellent, seven as good, two as moderate, and one as poor. The D‐TSF group performed significantly better than the Ilizarov group (95.24% *vs* 83.33%). Since the Johner–Wruhs Tibial Fracture Outcome Criteria assesses various aspects of a patient's performance, fracture deformity is the one of the most important aspects. As mentioned earlier, it is the ability of D‐TSF to simultaneously correct fracture deformities in multiple angles and planes that makes the residual deformities such as rotation, anterior/posterior tilt and other deformities of fracture end significantly better than those in the Ilizarov group. In conclusion, these findings suggest that TSF has clear advantages in adjusting fracture alignment and lower limb force lines. Moreover, patients who achieved good recovery of the lower limb force line could return to normal life following postoperative weight‐bearing functional exercise. However, the final follow‐up X‐ray analyses revealed no significant impact on the function of the affected limb in the two groups with excellent functional assessments, despite micro‐deformity at fracture end. Therefore, in the treatment of segmental tibial fractures, the emphasis should be on maximizing the lower limb force line to achieve functional repositioning instead of pursuing anatomical repositioning of fracture end. This approach can effectively promote the functional recovery of affected limb.

### Strengths and Limitations

The D‐TSF treatment is associated with minimal secondary damage to soft tissue, a straightforward and minimally invasive procedure, multiplanar stable fixation, and optimization of fracture alignment and lower limb force lines, therefore, it is highly effective therapeutic option for segmental tibial fracture. However, this study still has some limitations, including: (i) retrospective study design; (ii) a small sample size, and (iii) missing long‐term follow‐up data. Therefore, larger multicenter studies with longer follow‐up periods are needed to confirm the clinical efficacy of the D‐TSF in segmental tibial fracture fixation.

## Conclusions

D‐TSF external fixation is a reliable method to treat segmental tibial fracture with sever soft tissue injuries. It can provide multiplanar fracture stabilization, minimal soft tissue damage and early mobilization. More importantly, compared with Ilizarov, with the aid of computerized deformity and correction analysis systems, after surgery, D‐TSF allows for multiplanar adjustments, further improvement, further improvement in fracture alignment and lower limb force lines can be expected. The rate of fracture union is high, with minimal malalignment. Although needle tract infections are relatively common, they are uncomplicated and easily treated.

## Funding

This research was funded by the Natural Science Foundation Key Project of Tianjin (20JCZDJC00600) and Tianjin Health Research Project (TJWJ2023QN050).

## Conflict of Interest Statement

The authors declared no potential conflicts of interest with respect to the research, authorship, or publication of this article.

## Author Contributions

Guarantor of integrity of the entire study: Wei‐Guo Xu, Tao Zhang. Study concepts: Xun Sun. Study design: Qi‐Jun Zhao, Zhao Liu. Definition of intellectual content: Literature research: Qi‐Jun Zhao. Clinical studies: Zhao Liu, Xun. Experimental studies: Data acquisition: Xun Sun, Ning‐Ning Zhang. Data analysis: Xun Sun, Ning‐Ning Zhang. Statistical analysis: Zhao Liu. Manuscript preparation: Qi‐Jun Zhao, Zhao Liu. Manuscript editing: Qi‐Jun Zhao, Zhao Liu. Manuscript review: Wei‐Guo Xu, Tao Zhang. Financial support for research: Wei‐Guo Xu.

## Ethics Statement

Approval was obtained from the Medical Ethics Committee of Tianjin Hospital, No.: 2023 Medical Ethics Review 107 and all patients signed informed consent forms.
